# Cerebral Microbleeds May Be Less Detectable by Susceptibility Weighted Imaging MRI From 24 to 72 Hours After Traumatic Brain Injury

**DOI:** 10.3389/fnins.2021.711074

**Published:** 2021-09-30

**Authors:** Bálint S. Környei, Viktor Szabó, Gábor Perlaki, Bendegúz Balogh, Dorottya K. Szabó Steigerwald, Szilvia A. Nagy, Luca Tóth, András Büki, Tamás Dóczi, Péter Bogner, Attila Schwarcz, Arnold Tóth

**Affiliations:** ^1^Department of Medical Imaging, Medical School, University of Pécs, Pécs, Hungary; ^2^Department of Neurosurgery, Medical School, University of Pécs, Pécs, Hungary; ^3^MTA-PTE Clinical Neuroscience MR Research Group, Pécs Diagnostic Center, Pécs, Hungary; ^4^Neurobiology of Stress Research Group, Szentágothai Research Centre, University of Pécs, Pécs, Hungary; ^5^Department of Laboratory Medicine, Medical School, University of Pécs, Pécs, Hungary; ^6^MTA-PTE Clinical Neuroscience MR Research Group, Pécs, Hungary

**Keywords:** SWI MRI, traumatic brain injury, diffuse axonal injury, white matter, microbleeds, SWI, TMB

## Abstract

**Purpose:** A former rodent study showed that cerebral traumatic microbleeds (TMBs) may temporarily become invisible shortly after injury when detected by susceptibility weighted imaging (SWI). The present study aims to validate this phenomenon in human SWI.

**Methods:** In this retrospective study, 46 traumatic brain injury (TBI) patients in various forms of severity were included and willingly complied with our strict selection criteria. Clinical parameters potentially affecting TMB count, Rotterdam and Marshall CT score, Mayo Clinic Classification, contusion number, and total volume were registered. The precise time between trauma and MRI [5 h 19 min to 141 h 54 min, including SWI and fluid-attenuated inversion recovery (FLAIR)] was individually recorded; TMB and FLAIR lesion counts were assessed. Four groups were created based on elapsed time between the trauma and MRI: 0–24, 24–48, 48–72, and >72 h. Kruskal–Wallis, ANOVA, Chi-square, and Fisher’s exact tests were used to reveal differences among the groups within clinical and imaging parameters; statistical power was calculated retrospectively for each comparison.

**Results:** The Kruskal–Wallis ANOVA with Conover *post hoc* analysis showed significant (*p* = 0.01; 1−β > 0.9) median TMB number differences in the subacute period: 0–24 h = 4.00 (*n* = 11); 24–48 h = 1 (*n* = 14); 48–72 h = 1 (*n* = 11); and 72 h ≤ 7.5 (*n* = 10). Neither clinical parameters nor FLAIR lesions depicted significant differences among the groups.

**Conclusion:** Our results demonstrate that TMBs on SWI MRI may temporarily become less detectable at 24–72 h following TBI.

## Introduction

Traumatic brain injury (TBI) has become a devastating health problem in developed countries (Cole, 2004; Keenan and Bratton, 2006; Mathers and Loncar, 2006; Thompson et al., 2006; Chiu et al., 2007). TBI affects healthy, young, and often employed individuals, resulting in a heavy burden placed on society in both sociological and economic context (Berg et al., 2005; Lu et al., 2005; Keenan and Bratton, 2006; Tagliaferri et al., 2006; Thompson et al., 2006). Diffuse axonal injury (DAI) caused by shear forces due to acceleration and deceleration of brain compartments of different consistency during an accident is a common pathological factor regarding TBI (Moenninghoff et al., 2015; McGinn and Povlishock, 2016). DAI has been found in all severities of TBI and is referenced as an important determining factor regarding severity and outcome (Tang et al., 2012; Blennow et al., 2016). DAI encompasses a vast spectrum, dependent upon the severity and extent of injury, which can acutely manifest as immediate loss of consciousness or confusion resulting in a coma and/or cognitive dysfunction, or in other circumstances leads to reversible impairments to full axonal disruption (Gennarelli et al., 1986). A specific imaging marker regarding DAI will likely contribute to (1) early diagnosis and severity assessment, (2) timely onset of rehabilitation, (3) estimation of return to normal activity, (4) improved patient management, and (5) effectively following up on the patients’ condition and assuring the efficacy of the applied therapy (Paterakis et al., 2000; Wallesch et al., 2001). Currently, DAI is considered an exclusionary diagnosis; conventional imaging techniques are considered not to be sensitive enough to fully visualize it (Gennarelli et al., 1986). Certain modern MRI techniques however are capable of detecting pathological components regarding DAI (Blitstein and Tung, 2007; Sharma et al., 2018).

Functional MRI, diffusion tensor imaging (DTI), or MR spectroscopy promises a comprehensive understanding of DAI; however, these methods are mostly applicable in the form of statistical group analysis. To date, their individual routine clinical application is not entirely clarified (Kumar et al., 2009; Asano et al., 2012; Toth et al., 2013). T2 × MRI techniques—sensitive in visualizing magnetic susceptibility—are capable of visualizing microscopic bleeding; among them, susceptibility weighted imaging (SWI) is reported to be the most sensitive (Haacke et al., 2009; Mittal et al., 2009; Cheng et al., 2013).

By definition, traumatic microbleeds (TMBs) in SWI appear as ovoid or curvilinear hypointensities localized in the white matter (WM), mostly at the WM–gray matter (WM-GM) junction, in the brainstem, or in the corpus callosum and the region of the basal ganglia. Imaging of TMBs is indeed challenging: their visibility and number are influenced by numerous clinical and technical factors (e.g., age, SWI field strength, SWI slice thickness, TBI severity, and neurological comorbidities) (Parizel et al., 2001; Ripoll et al., 2003; Greenberg et al., 2009).

Although TMBs are reportedly potential markers of DAI (Di Ieva et al., 2015), there is a lack of consensus regarding how DAI exactly relates to hemorrhagic lesions. A DTI study implies that DAI may develop without focal MRI lesions in TBI (Kumar et al., 2009) and that DTI is also capable of revealing minute lesions of the WM and deep brain structures, which may not be visualized on T2 × GRE or fluid-attenuated inversion recovery (FLAIR) images (Asano et al., 2012; Spitz et al., 2013). According to an increasing number of studies, hemorrhagic lesion localization seemingly is more important than the overall number associated with DAI severity assessment (Toth et al., 2018; Andreasen et al., 2019). Based on histological analysis of one patient, a very recent study suggests DAI does not co-localize with TMBs (Griffin et al., 2019). Nevertheless, nearly all studies concur that a certain number, form, or localization of TMBs are associated with more severe injuries and less favorable outcomes; therefore, their detection is of clinical importance (Beauchamp et al., 2013; Kim and Lee, 2013; Yuh et al., 2013; Akoudad et al., 2016; de Haan et al., 2017; van der Horn et al., 2018). Interestingly, some human case studies reported significant temporal changes regarding TMB morphology in the acute to subacute phase following injury, yet it was unclear if these changes mean only changes in appearance, or true biophysical–biochemical changes in reference to the hemorrhages (Ezaki et al., 2006; Kallakuri et al., 2015; Toth et al., 2016; Watanabe et al., 2016; Lawrence et al., 2017).

In our recent study, we managed to better understand this phenomenon based on a rodent cerebral microbleed model: surgically created artificial microscopic WM bleedings showed a significant and transient intensity increase (i.e., decrease in visibility) between 24 and 96 h following surgery. Additionally, 69% of the lesions became “invisible,” i.e., isointense to the WM, which was followed by a reappearance. Histology confirmed that microbleeds were present at every time point when MRI measurements were made; therefore, we regarded this phenomenon to be due to changes in biophysical properties of microbleeds. We concluded that the timing of SWI may be critical to avoid false-negative results (Tóth et al., 2019). Additionally, the relative inconsistency in previous studies regarding the clinical applicability of SWI MRI in TBI may be explained by our findings. In the present study, we aimed to reveal if such transient reduction in TMB visibility occurs in humans as well, and we aimed to define the typical time frame of this phenomenon.

## Materials and Methods

### Subjects

**FIGURE 1:**
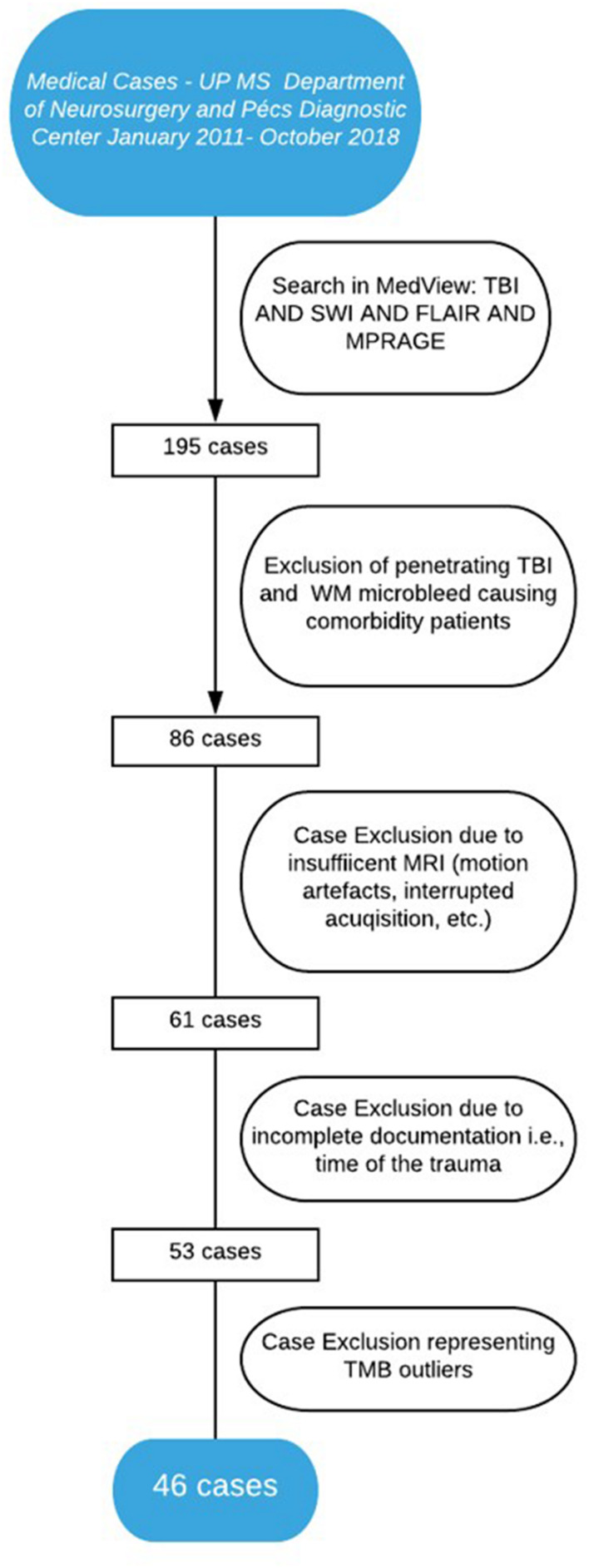
Algorithm of patient inclusion.

A total of 195 adults with closed TBI, compliant to our MRI protocol, were initially included retrospectively from a prospectively collected observational cohort at UP Clinical Center Department of Neurosurgery and Pécs Diagnostic Center. A crucial criterion was precise TBI time documentation. Additionally, the exact time of admission, and CT and MRI acquisition were also recorded. Exclusion criteria included any diagnoses of comorbidities capable of causing WM TMBs [e.g., fat embolism, chronic hypertension, cerebral amyloid angiopathy, cavernous malformations, epilepsy, Alzheimer’s disease, dementia or migraine, brain tumor, or cerebral metastasis (Susman et al., 2002; Nakata-Kudo et al., 2006; Brain Trauma Foundation et al., 2007; Haacke et al., 2007; Oh et al., 2008; Ayaz et al., 2010; Beauchamp et al., 2013; Kim and Lee, 2013; Nakagami et al., 2014; Shams et al., 2015; Yamauchi et al., 2016; Trifan et al., 2017)] based on patient medical records. Grubbs’ test was applied to exclude patients with outlier TMB numbers. Figure 1 shows our algorithm and criteria of inclusion and exclusion.

The final number of patients eventually was narrowed to 46 cases who were eligible for the study [37 male and nine female; 6 symptomatic, eight mild, and 32 severe according to the Mayo Clinic Classification of Traumatic Brain Injury (Malec et al., 2007)]. Investigations were carried out compliant to the rules of the Declaration of Helsinki, and ethical approval was granted from the Institutional Review Board of the University of Pécs (No. 4525). Written informed consent was obtained from all the participants or their legally authorized representatives regarding the MRI scans used in the study.

### Clinical Data and Admission CT Parameters

Traumatic brain injury severity was individually defined according to the Mayo Clinic Classification of Traumatic Brain Injury (symptomatic, mild, and moderate–severe) (Malec et al., 2007). Age at the time of trauma, gender, Rotterdam (Maas et al., 2005) and Marshall CT scores (Marshall et al., 1992) (assessed on admission CT), MRI field strength (1.5 or 3 T), FLAIR lesion number, and macroscopic injuries were recorded. Furthermore, the total approximate volume of contusions was recorded on admission, through individual CTs (MedView^TM^) in accordance to the following formula developed by Kothari et al. (1996; Tables 1–3):


$$\mathrm{CV}=\frac{\mathrm{LPD}\times \mathrm{NSL}\times \mathrm{SL}}{2}$$

**TABLE 1:** Age, causes, and symptoms of TBI according to admission data.

			Σ	Groups			
				0–24 h	24–48 h	48–72 h	72 h <
**Number of patients**			46	11	14	11	10

**Median age for whole set of patients, mean for groups in years**			50 (IQR 27–67)	34.45 (SD = 25.72)	52.00 (SD = 25.45)	53.91 (SD = 18.65)	42.00 (SD = 24.59)
Causes of TBI		Falls	21	2	9	5	5
		Traffic accident	15	6	2	3	4
		Violence	3	1	0	2	0
		Other*	7	2	3	1	1
Symptoms of tBI	*Physical symptoms*	Nausea/vomiting	11	2	2	4	3
		Amnesia	9	1	2	5	1
		Headache	7	2	0	3	2
		Loss of consciousness	6	1	1	3	1
		Somnolence	2	1	0	0	1
		Dizziness	2	1	0	1	0
	Sensory symptoms**		2	1	0	1	0
	History could not be obtained		12	3	3	3	3
	Other***		7	3	1	1	2
	Asymptomatic		6	0	5	0	1

*TBI, traumatic brain injury; PTSD, post-traumatic stress disorder.*

*Unknown, intoxicated, GM, sports, etc.

**For example, blurred or double vision, numbness, hearing impairment, etc.

***Disorientation, agitation, seizures, PTSD, and thoracic emphysema all occurred.

where CV is the contusion volume, LPD is the product of the longest perpendicular diagonals of the contusion appearing on admission CT, NSL is the number of slices on which the contusion is present, and SL is slice thickness.

### MRI Acquisition

Susceptibility weighted imaging, T1-weighted MPRAGE and FLAIR images were assessed. Brain MRI was performed using 1.5T (Avanto/Avantofit) and 3T (Magnetom Trio/Prisma Fit) Siemens (Munich, Germany) MR scanners; and, in the case of SWI, special attention was given to the evaluation of MRI images with higher field strength and thinner slices in the estimated time frame of TMB disappearance (24–72 h) as shown in Table 2.

**TABLE 2:** Influential factors of TMB count: age, TBI severity, and relevant SWI imaging data, level of significance of differences between groups.

		Σ	*Groups*				Significance
			0–24 h	24–48 h	48–72 h	72 h <	
**Number of patients**		46	11	14	11	10	

Median age for whole set of patients, mean for groups in years*		50 (IQR 27–67)	34.45 (SD = 25.72)	52.00 (SD = 25.45)	53.91 (SD = 18.65)	42.00 (SD = 24.59)	*p* = 0.19
Gender***	Male	37	10	10	9	8	*p* = 0.72
	Female	9	1	4	2	2	
TBI severity (MAYO)***	Symptomatic	6	0	3	3	0	*p* = 0.11
	Mild	8	3	0	3	2	
	Moderate-severe	32	8	11	5	8	
MARSHALL score***	I	13	4	5	2	2	*p* = 0.73
	II	8	2	2	1	3	
	III	8	2	1	4	1	
	IV	0	0	0	0	0	
	V	0	0	0	0	0	
	VI	17	3	6	4	4	
Rotterdam score***	1	27	6	12	4	5	*p* = 0.09
	2	14	3	1	7	3	
	3	2	1	0	0	1	
	4	2	1	0	0	1	
	5	0	0	0	0	0	
	6	0	0	0	0	0	
SWI field strength***	1.5 T	11 (23.91%)	4 (36.36%)	3 (21.43%)	2 (18.18%)	2 (20.00%)	*p* = 0.77
	3 T	35	7	11	9	8	
SWI slice thickness (mm)***	1.15	1	1	0	0	0	*p* = 0.59
	1.2	1	1	0	0	0	
	1.5	32	5	10	9	8	
	2	8	2	2	2	2	
	3	3	2	1	0	0	

Results of one-way ANOVA (*) and Fisher’s exact test (***).

*TMB, traumatic microbleed; TBI, traumatic brain injury; SWI, susceptibility weighted imaging.*

T1-weighted high-resolution images were obtained using a three-dimensional (3D) magnetization-prepared rapid gradient-echo (MP-RAGE) sequence [inversion time (TI) = 900 ms; repetition time (TR) = 1,400 ms; echo time (TE) = 3 ms; slice thickness = 1.0 mm; field of view (FOV) = 192 mm × 256 mm; matrix size = 192 × 256. 3D and 2D FLAIR images were acquired using the following: TI = 1,888.1–2,713.4 ms; TR = 5,000–8,910 ms; slice thickness = 4.0 mm; FOV = 192–225 mm × 225–256 mm; matrix size = 187–384 × 256–512. 3D SWI images were acquired as follows: TR = 46–49 ms; TE = 40 ms; slice thickness = 2.0–3.0 mm; FOV = 158—01 mm × 230 mm; matrix size = 137–177 × 192–256, with no inter-slice gap for 1.5 T and (3D) MP-RAGE sequence (TI = 900 or 1,100 ms; TR = 1,380 or 2,530 ms; TE = 2.2 or 3.4 ms; slice thickness = 1.0 or 1.1 mm; FOV = 211 or 256 mm × 211 or 256 mm; matrix size = 192 or 256 × 192 or 256. 3D and 2D FLAIR images were acquired using the following: TI = 1,800–2,500 ms; TR = 5,000–9,000 ms; slice thickness = 0.9–4.0 mm; FOV = 193–230 mm × 220 or 230 mm; matrix size = 192–512 × 256 or 512. 3D SWI images were acquired as follows: TR = 27 ms; TE = 20 ms; slice thickness = 1.5 mm; FOV = 158–199 mm × 220 or 230 mm; matrix size = 167–223 × 256, with no inter-slice gap for 3T measurements (Supplementary Table 1).

Elapsed time expressed as hours between the trauma and the nearest SWI imaging was recorded as follows: time of the trauma was registered according to admission documentation, recorded by the National Ambulance Service or the Emergency Department of UP MS, and the exact time of scans was documented from the MRI scans’ DICOM data.

### Hemorrhagic and Nonhemorrhagic MRI Lesion Detection

Anonymized CT and MRI scans were read by AT and BK, with both authors having more than 6 years of experience in human brain CT and MRI data processing, blinded to clinical and time-to-scan data. Final lesion counts were described as per agreement. Lesion parameters were validated by PB, who specializes in neuroradiology with more than 10 years of experience.

Susceptibility weighted imaging TMBs were defined as ovoid or curvilinear hypointensities localized in the WM, mostly at the WM-GM junction, in the brainstem, or in the corpus callosum and the region of the basal ganglia. For precise TMB identification, exclusion of SWI lesion mimics (intersects of veins, bottom of sulci, calcium deposits, artifacts caused by air–tissue interfaces, or macroscopic bleeding caused by, e.g., an intraventricular drain) had to be performed. Therefore, SWI images were registered with high-resolution T1-weighted images using FMRIB’s Linear Image Registration Tool (FLIRT), which allowed a multimodal and anatomically accurate assessment of TMBs (Jenkinson and Smith, 2001; Jenkinson et al., 2002; Greve and Fischl, 2009).

Lesions adjacent to contusions, intraventricular hemorrhage, or bone–air interface artifacts (e.g., near mastoid process) or an external ventricular drain were excluded. The overall TMB number and localization according to Adams et al. (1989) was individually recorded.

Fluid-attenuated inversion recovery lesions were defined as focal, round to ovoid hyperintensities and strictly localized within the WM.

**FIGURE 2:**
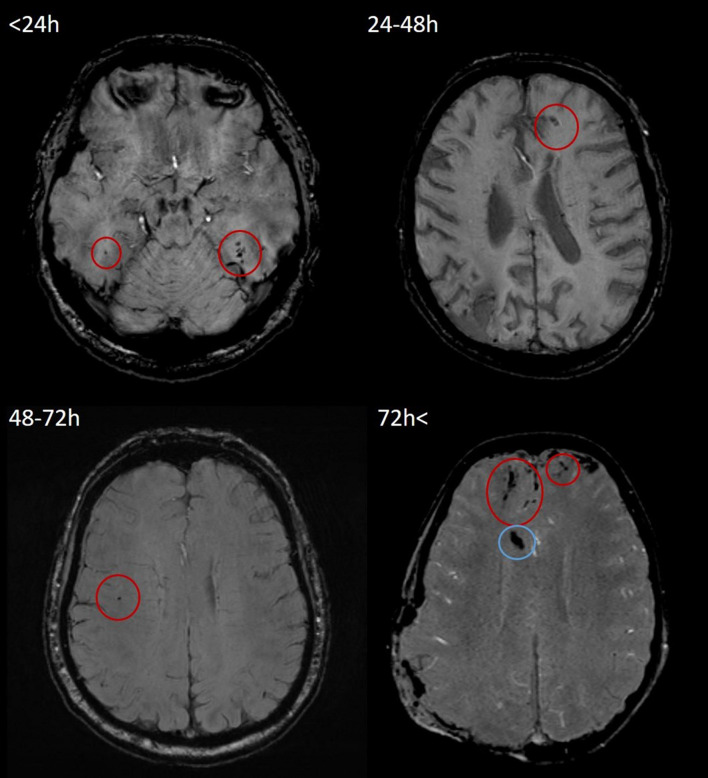
Representative examples of TMBs in SWI images in <24, 24–48, 48–72, and 72 h < groups. All four SWI measurements were performed on a 3T Siemens Magnetom Prisma MRI scanner. According to Mayo Clinic Classification, both cases (top left, 21-year-old male; top right, 50-year-old male; bottom left, 64-year-old male; and bottom right, 60-year-old male) were classified as severe TBI. TMBs are indicated by red circles. In the bottom left image, hypointensity caused by the intraventricular drain is indicated by a blue circle. TMB, traumatic microbleed; SWI, susceptibility weighted imaging; TBI, traumatic brain injury.

**FIGURE 3:**
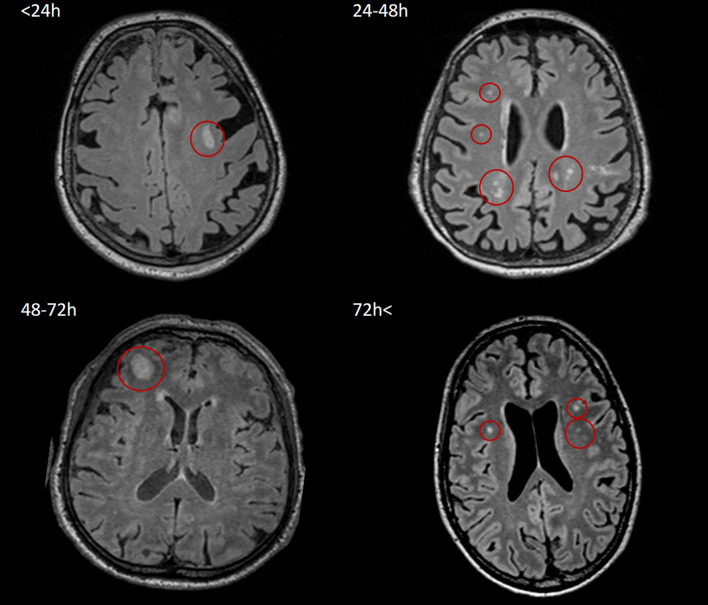
Representative images of nonhemorrhagic/FLAIR lesions in <24, 24–48, 48–72, and 72 h < groups. All four FLAIR measurements were performed on a 3T Siemens Magnetom Prisma MRI scanner. According to Mayo Clinic Classification, two of the four patients (top right, 77-year-old female; bottom left, 31-year-old male) suffered symptomatic TBI, and two of them (top left, 75-year-old female; bottom right, 27-year-old male) were classified as severe TBI; lesions are indicated by red circles. FLAIR, fluid-attenuated inversion recovery; TBI, traumatic brain injury.

Examples of SWI and FLAIR lesions at different time points are shown in Figures 2, 3.

### Statistical Analysis

MedCalc for Windows, version 19.1.1 (MedCalc Software, Ostend, Belgium) was used regarding all statistical analyses on the anonymized data except for Fisher’s exact test, which was processed using the IBM SPSS Statistics for Windows, Version 25.0 (IBM Corp., Armonk, NY, United States). Descriptive statistics were applied to summarize clinical, CT, and MRI data. In cases of non-normal distributed data median and the interquartile range, and in cases of normally distributed data, mean and SD are depicted in Table 2.

To model temporal trends of lesions, linear, exponential, and second-degree polynomial trend lines were aligned to the number of SWI TMBs and FLAIR hyperintensities in function of elapsed time following TBI; Grubbs’ test was applied to exclude outliers. For further analysis, the best-fitting trend line (the one with the highest *R*^2^ value) was selected. For both TMBs and FLAIR lesions, a second-order polynomial trend line aligned the best (*R*^2^ = 0.20). The solution of this trend line’s equation regarding the average TMB count defined the exact time frame in which TMB numbers were below average.

The commonly referred defined time frame was adapted considering clinical and practical applicability; thus, four groups were created based on the elapsed time between the trauma and the earliest MRI: 0–24 h (*n* = 11); 24–48 h (*n* = 14); 48–72 h (*n* = 11); and 72 h < (*n* = 10). The Shapiro–Wilk normality test was applied to test the distribution of TMB, and FLAIR lesion numbers, age, contusion number, and total volume. Fisher’s exact test with continuity correction was used to elucidate differences in occurrence of categorical variables between the groups, possibly affecting lesion count such as gender, Mayo Clinic TBI classification, Rotterdam and Marshall scores, TMB localization, slice thickness, and scanner field strength. The Kruskal–Wallis ANOVA with Conover *post hoc* test was applied to assess the average TMB and FLAIR lesion count, contusion number, and volume differences between the groups; statistical power of the comparisons was calculated with R Statistical Software’s MultNonParam-kwpower package (version 3.6.0.; R Foundation for Statistical Computing, Vienna, Austria).

## Results

**FIGURE 4:**
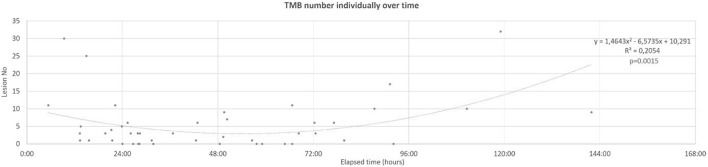
Individual TMB number over time, fitted second-order polynomial trend line. TMB, traumatic microbleed.

According to the Mayo Clinic Classification System regarding TBI, severity distributed was as 6 = symptomatic, 8 = mild, and 32 = moderate–severe in the set of 46 patients. The distribution of age in our entire set of patients was not normally distributed (*p* = 0.02); mean age in time of the trauma was 46.09 (SD = 24.39) years. A total of 248 TMBs (131 on 3-T and 117 on 1.5-T scanners) and 220 hyperintense focal lesions in FLAIR were identified among 46 patients. In reference to acute CTs, 16 contusions were detectable in nine of our patients. Detailed demographic and admission clinical data are presented in Tables 1–3. A second-order polynomial trend line is depicted regarding the individual TMB number over time with the highest *R*^2^ value. In reference to the TMB number *R*^2^ = 0.2, *p* = 0.002, *y* = 3.0206X^2^ − 13.065X + 15.04 values were yielded (Figure 4). The average TMB number with respect to the entire population was 5.4. Substituting this value in the quadratic formula:


$$x\,1;2\,=\,\frac{-b\pm \sqrt{b^{2}-4\,a\,c}}{2\,a}$$

**FIGURE 5:**
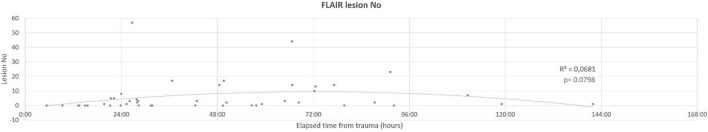
Individual FLAIR lesion number over time, representing the second-order polynomial trend line. FLAIR, fluid-attenuated inversion recovery.

X_1_ = 85 h 55 min and X_2_ = 21 h 50 min were yielded. The nearest two acquisitions in our set of patients to these results were 21 h 11 min and 79 h 45 min following trauma. This result supported a strong tendency regarding the further division of our data into the groups described in methods [0–24 h (*n* = 11); 24–48 h (*n* = 14); 48–72 h (*n* = 11) 72 h < (*n* = 10)]. Additionally, a polynomial tendency line was represented with the highest *R*^2^ value for FLAIR lesion numbers (*R*^2^ = 0.07, *p* = 0.08, Figure 5).

**FIGURE 6:**
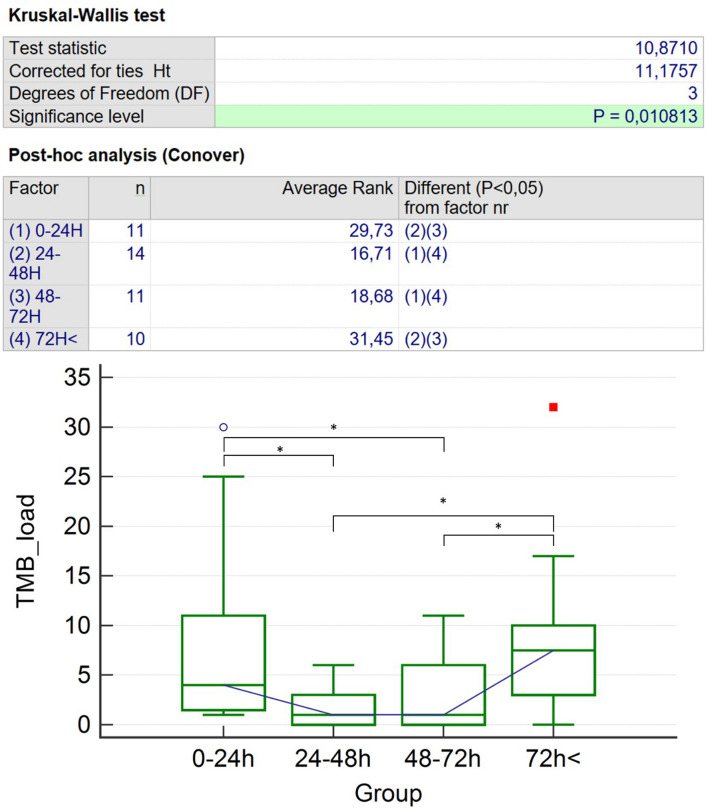
Kruskal–Wallis with Conover *post hoc* test: results for TMB number differences. The symbol “*” represents significant (*p* < 0.05) differences of TMB count; the blue circle and the red square stand for the two patients with the highest TMB count. TMB, traumatic microbleed. * represents significant (*p* < 0.05) differences of TMB count.

The Shapiro–Wilk normality test revealed that both TMB (0–24 h, *p* = 0.003; 24–48 h, *p* = 0.005; 48–72 h, *p* = 0.003; and 72 h <, *p* = 0.04) and FLAIR lesion count significantly differed from normal distribution in every group (0–24 h, *p* = 0.003; 24–48 h, *p* = 0.004; 48–72 h, *p* = 0.003; and 72 h <, *p* = 0.04) and in the entire population, as well (*p* < 0.001 for both TMB and FLAIR lesion count). Contusion numbers did not show normal distribution (*p* < 0.001 in every group); contusion volumes as continuous variables also failed to show normal distribution; median contusion volumes were 0–24 h = 842.00 (IQR 539.29–1,316.00) mm^3^; 24–48 h = 331.50 (IQR 0.00–1,642.25) mm^3^; 48–72 h = 214.00 (IQR 143.28–9,480.25) mm^3^; 72 h ≤ 129.60 mm^3^. Patients’ age in each group did not significantly differ from that which is normally distributed: 0–24, *p* = 0.12; 24–48 h, *p* = 0.16; 48–72 h, *p* = 0.28; and 72 h <, *p* = 0.14. Results for comparison of clinical and CT data among groups were as follows: mean age in years were 0–24 h = 34.45 (SD = 25.72); 24–48 h = 52.00 (SD = 25.45); 48–72 h = 53.91 (SD = 18.65); and 72 h ≤ 42.00 (SD = 24.59). One-way ANOVA revealed that there were no significant differences in relation to age, *p* = 0.19 (Table 2). Fisher’s exact test did not reveal significant differences with respect to the Mayo Clinic TBI classification (*p* = 0.11), Rotterdam (*p* = 0.09) and Marshall (*p* = 0.73) scores, SWI field strength (*p* = 0.77), and slice thickness (*p* = 0.59), in the distribution of macroscopic pathologies (*p* = 0.79) or the gender of our patients (*p* = 0.72) (Table 2). Median TMB count in each group was as follows: 0–24 h = 4.0 (IQR 1.50–11.00); 24–48 h = 1.0 (IQR 0.00–3.00); 48–72 h = 1.0 (IQR 0.00–6.00); and >72 h = 7.5 (IQR 3.00–10.00). Median FLAIR lesion count was 0–24 h = 0.00 (IQR 0.00–1.75); 24–48 h = 0.50 (IQR 0.00–14.00); 48–72 h = 3.00 (IQR 1.00–4.00); and >72 h = 5.00 (IQR 1.00–14.00) (Table 3). The Kruskal–Wallis test for TMBs revealed significant differences (*p* = 0.01) between the groups but showed no significant correlations with respect to FLAIR lesions (*p* = 0.18) and number of contusions (*p* = 0.66) or in respect to the average contusion volume (*p* = 0.69), as it is shown in Table 3 and Figure 6. Statistical power was 1 − β > 0.9 for TMB, FLAIR lesion count, and contusion volume comparisons. TMB localization did not show differences among the groups (*p* = 0.68).

**TABLE 3:** Traumatic microbleeds count and localization, macroscopic pathologies, FLAIR lesion counts, contusion number, and volume and the level of significance of differences between groups.

Number of patients		Σ	*Groups*				Significance
			0–24 h	24–48 h	48–72 h	72 h <	
		
		46	11	14	11	10	
TMB load***	Total	248	95	26	33	94	***p* = 0.011**
	Median	3.00 (IQR 0.00–7.00)	4.00 (IQR 1.50–11.00)	1.00 (IQR 0.00–3.00)	1.00 (IQR 0.00–6.00)	7.50 (IQR 3.00–10.00)	
TMB localization***	Subcortical	220	85	25	27	83	*p* = 0.68
	Corpus callosum	19	7	1	3	8	
	Brainstem	9	3	0	3	3	
FLAIR lesion Number***	Total	277	20	124	32	101	*p* = 0.18
	Median	2.00 (IQR 0.00–7.25)	0.00 (IQR 0.00–1.75)	0.50 (IQR 0.00–14.00)	3.00 (IQR 1.00–4.00)	5.00 (IQR 1.00–14.00)	
Contusion Number***	Total	16	7	3	5	1	*p* = 0.66
	Median	0.00 (IQR 0.00–0.75)	0.00 (IQR 0.00–1.50)	0.00 (IQR 0.00–0.00)	0.00 (IQR 0.00–1.00)	0.00 (IQR 0.00–0.00)	
Contusion volume***	Total	19837.8	2741.00	4064.50	12902.7	129.60	*p* = 0.69
	Median	378.25 (IQR 124.65–1446.00)	842.00 (IQR 539.29–1316.00)	331.50 (IQR 0.00–1642.25)	214.00 (IQR 143.28–9480.25)	129.60	
Macroscopic pathologies***	Intraventricular hematoma	2	1	0		1	*p* = 0.79
	Skull fracture	13	5	5		3	
	Epidural hematoma	3	3	0		0	
	Subdural hematoma	7	1	3		3	
	Subarachnoideal hematoma	7	2	4		1	
	Atrophy	4	1	3		0	

Results of the Kruskal–Wallis with Conover *post hoc* test (**) and Fisher’s exact test (***).

*TMB, traumatic microbleed; FLAIR, fluid-attenuated inversion recovery.*

## Discussion

This retrospective study on cross-sectional imaging data enabled an indirect validation of the phenomenon of general transient TMB visibility decrease in human SWI scans. A trend line representing the individual TMB count revealed a nadir between approximately 21 and 80 h following trauma. According to practical considerations, these time points were adjusted to 24 and 72 h for further analysis. Due to the cross-sectional nature of the study, it was crucial to check the presence regarding factors potentially posing as a bias. TBI severity (according to Mayo Clinic Classification and Marshall score), distribution of macroscopic pathologies, SWI field strength, age, gender distribution, or any of the influential factors among the time groups significantly did not differ. Thus, these time groups proved ideally suitable to examine the influence of elapsed time between TBI and SWI on TMB visibility. Median TMB count in the 24–72 h period was significantly lower than in the hyperacute (0–24 h) period or than in the 72 h < period. Although TMB formation is reported to be significantly more frequent among older patients, we experienced lower median TMB numbers in groups in which the average age was higher.

As an internal control of our study, we examined the occurrence of FLAIR lesions, as markers of edema developing along with DAI, over time. Distinctly, FLAIR lesion count did not significantly differ in the examined time period, which suggests that we are confronting a phenomenon specific for TMBs. FLAIR lesions are also regarded as markers of DAI and injury severity and may be more stable over the acute to subacute phase; however, previous studies suggest that they are not so specific and clearly related to the extent of actual DAI and prognosis (Marquez De La Plata et al., 2007; Ding et al., 2008; Bigler et al., 2013; Amyot et al., 2015) as TMBs (Tong et al., 2003).

The findings of this study are congruent with our former results: in our rat model, TMBs showed significant temporal visibility reduction in SWI; they often became completely invisible in the 24–96 h period, while microbleeds’ consistent presence was histologically proven. Reappearance was demonstrated after 96 h. In this article, the authors expressed that the most possible explanation regarding acute TMB disappearance may be clot retraction caused by voxel level homogenization resulting in signal gain. The authors also suspected the possible role of methemoglobin formation and consequential T1 shine through. The reappearance of microbleeds could be explained by the development of late breakdown products of hemoglobin as hemosiderin and ferritin, known to be superparamagnetic (Bradley, 1993; Tóth et al., 2019).

Our findings support former case studies reporting TMBs’ morphological changes in SWI, which, moreover coincide with case observations by Watanabe et al. that TMB invisibility may occur roughly between 24 h and 7 days after formation (Watanabe et al., 2016). Furthermore, in a study focusing on cerebral blood flow changes in an experimental closed head injury rat model, the authors reported some ancillary cases in which hypointense foci congruent with TMBs disappeared and later reappeared (Kallakuri et al., 2015).

The main practical consequence of these results implies that SWI may be false-negative for TMBs between 24 and 72 h following injury. Half of our patients (23 of 46) were examined in this time period. This demonstrates at least in our institution that there is a considerable chance for patients being MRI scanned within the “decreased TMB visibility” period. We assume that this may be a general problem, since MRI is almost always electively, secondarily performed to admission CTs, often after clinical stabilization. Additionally, our finding may be applicable in relation to not only TMBs but also the acute examination of every pathology capable of causing WM TMBs. Although 1.5T and 3T field strength acquisition rates were rather evenly distributed among time points, considering overall lesion counts, 3T detected somewhat more lesions (131) than 1.5T (117), supporting the fact that 3T has a higher sensitivity for TMBs irrespective of imaging timing.

The main limitations of this study are the limited sample size, as a result of our strict inclusion criteria; and temporal features of TMBs were indirectly investigated based on cross-sectional data. Also, according to the assumed nature of temporal changes of TMB visibility, there could be an uncertainty of TMB development in patients examined between 24 and 72 h. Direct investigation of the temporal visibility changes of TMBs would have been only possible by a longitudinal study. Unfortunately, the implementation of multiple time point follow-up MRI studies in TBI, especially when including severely injured patients, is almost impossible: although MRI itself can be regarded as a safe imaging technique, the relatively long acquisition time can be inconvenient for TBI patients or may even pose risk for severely injured patients due to patient and anesthesiological/intensive care gear transportation. However, very strict patient selection criteria were applied, and factors most possibly affecting TMB presence were considered to minimize biased results.

## Conclusion

This retrospective study indirectly substantiates that short-term temporary TMB visibility decrease is generally present not only in rodents but in humans as well. Based on our results, TMB visibility decrease seems to occur from 24 to 72 h following TBI. MRI for detecting TMBs in this period may result in false-negative findings, leading to an under-diagnosis of injury severity and false prognosis estimation.

## Data Availability Statement

The raw data supporting the conclusions of this article will be made available by the authors, without undue reservation.

## Ethics Statement

The studies involving human participants were reviewed and approved by the Institutional Review Board of the University of Pécs (No. 4525). The patients/participants provided their written informed consent to participate in this study.

## Author Contributions

BK: study conception and design, data acquisition, analysis and interpretation of the data, drafting, and final approval. VS: study design, data acquisition, draft revision, and final approval. GP, SN, AS and AT: study conception and design, analysis and interpretation of the data, draft revision, and final approval. BB and DS: analysis and interpretation of the data, draft revision, and final approval. LT: data acquisition, draft revision, and final approval. AB, TD, and PB: conception and design, draft revision, and final approval. All authors agreed to be accountable for all aspects of the work in ensuring that questions related to the accuracy or integrity of any part of the work are appropriately investigated and resolved.

## Conflict of Interest

The authors declare that the research was conducted in the absence of any commercial or financial relationships that could be construed as a potential conflict of interest.

## Publisher’s Note

All claims expressed in this article are solely those of the authors and do not necessarily represent those of their affiliated organizations, or those of the publisher, the editors and the reviewers. Any product that may be evaluated in this article, or claim that may be made by its manufacturer, is not guaranteed or endorsed by the publisher.

## Supplementary Material

Supplementary Figure 1 TMB count as a function of time in patients scanned by 1.5T (*n* = 11) or 3T (*n* = 35) scanners (*y*-axis: TMB count; *x*-axis: elapsed time between trauma and MRI scan individually). A 2nd order polynomial trend line could be fitted with the highest *R*^2^ value [*R*^2^ = 0,1318 (1.5T) and R^2^ = 0,2261 (3T)] on individual TMB count in the same manner as when patients scanned with two different field strength were examined combined.

Supplementary Figure 2 In our final 46 patients included, there were only two cases -of which one is shown in this figure- when a TMB (indicated by red arrow) and a non haemorrhagic FLAIR lesion (indicated by blue arrows) were co-localised.

Supplementary Table 1 MRI measurement parameters for each applied protocols.

## Abbreviations

DAI: diffuse axonal injury
FA: fractional anisotropy
FA-SPM: fractional anisotropy images analyzed by statistical parametric mapping
FLIRT: FMRIB’s Linear Image Registration Tool
TBI: traumatic brain injury
TMB: traumatic microbleed
UP MS: University of Pécs Medical School.
