# Supplementary figures and images for: Cerebral Microbleeds May Be Less Detectable by Susceptibility Weighted Imaging MRI From 24 to 72 Hours After Traumatic Brain Injury
Source: Front Neurosci. 2021 Sep 30;15:711074. doi: 10.3389/fnins.2021.711074 (PMC8514822; doi:10.3389/fnins.2021.711074)

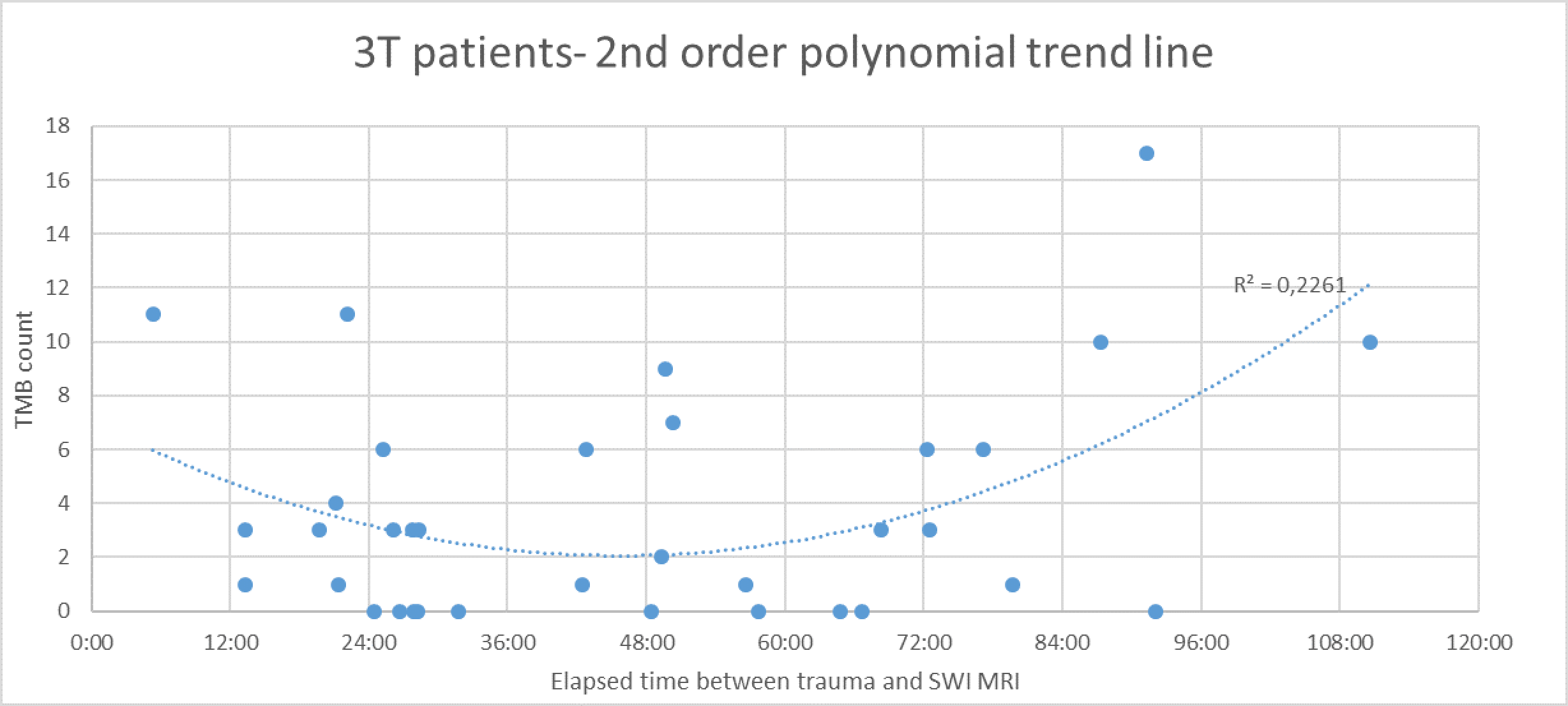

Supplement: Supplementary Figure 1 — TMB count as a function of time in patients scanned by 1.5T (n = 11) or 3T (n = 35) scanners (y-axis: TMB count; x-axis: elapsed time between trauma and MRI scan individually). A 2nd order polynomial trend line could be fitted with the highest R2 value [R2 = 0,1318 (1.5T) and R2 = 0,2261 (3T)] on individual TMB count in the same manner as when patients scanned with two different field strength were examined combined. [file Image_1.png]

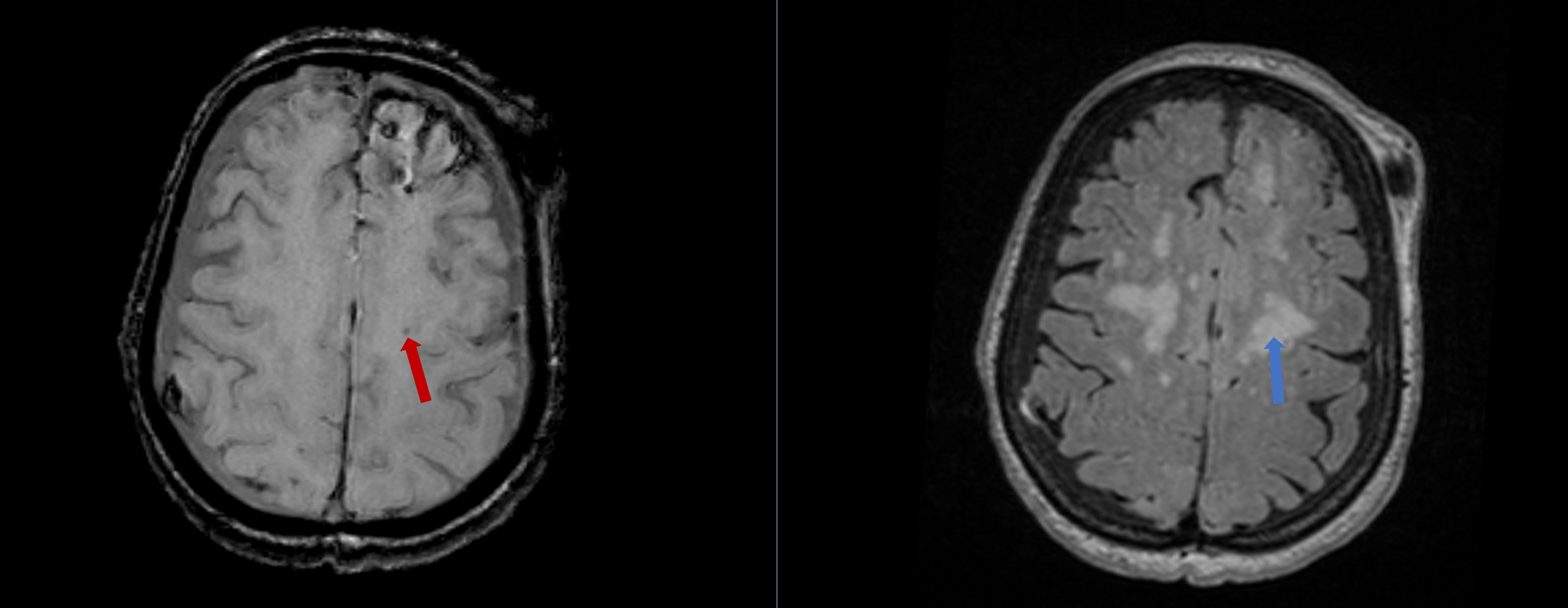

Supplement: Supplementary Figure 2 — In our final 46 patients included, there were only two cases -of which one is shown in this figure- when a TMB (indicated by red arrow) and a non haemorrhagic FLAIR lesion (indicated by blue arrows) were co-localised. [file Image_2.jpeg]
