# Supplementary material for: Cerebral Microbleeds May Be Less Detectable by Susceptibility Weighted Imaging MRI From 24 to 72 Hours After Traumatic Brain Injury
Source: Front Neurosci. 2021 Sep 30;15:711074. doi: 10.3389/fnins.2021.711074 (PMC8514822; doi:10.3389/fnins.2021.711074)
Supplement: Supplementary Table 1 — MRI measurement parameters for each applied protocols. [file Table_1.pdf]

MRI scanner / number of patients measures with protocol	T2					MPRAGE						FLAIR						SWI				
	TR	TE	SL	FOV	matrix	TI	TR	TE	SL	FOV	matrix	TI	TR	TE	SL	FOV	matrix	TR	TE	SL	FOV	matrix
**3T Prisma / 31**	**5500**	**106**	**4**	**220*220**	**320*320**	**1100**	**2530**	**3.4**	**1**	**256x256**	**256x256**	**) (3D space,**	**5000**	**387**	**0.9**	**230x230**	**512x512**	**27**	**20**	**1.5**	**199x220**	**223x256**
1,5T Avanto / 1	6220	84	5	192*256	192*256	900	1400	3	1	192*256	192*256	1888.1	5000	99	4	192*256	384*512	46	40	3	172*230	137*192
1,5T Avanto / 1	6651	97	4	188*225	268*320	900	1400	3	1	192*256	192*256	2872.1	8750	93	4	225*225	230*256	49	40	2	180*230	158*256
1,5T Avanto / 1	6651	97	4	188*225	268*320	900	1400	3	1	192*256	192*256	2713.4	8910	93	4	195*240	187*256	49	40	2	165*230	145*256
1,5T Avanto / 1	6651	97	4	188*225	268*320	900	1400	3	1	192*256	192*256	2713.4	8910	93	4	225*225	230*256	49	40	2	180*230	158*256
1,5T Avanto / 1	6821	97	4	188*225	268*320	900	1400	3	1	192*256	192*256	2710.4	8890	93	4	225*225	230*256	49	40	2	187*230	164*256
1,5T Avanto / 1	7270	97	4	188*225	268*320	900	1400	3	1	192*256	192*256	2713.4	8910	93	4	225*225	230*256	49	40	2	201*230	177*256
1,5T Avanto / 1	7460	84	5	192x256	192x256	900	1400	3	1	192*256	192*256	1888.1	5000	99	4	192*256	384*512	46	40	3	172*230	137*192
1,5T Avanto / 1	7954	84	5	192*256	192*256	900	1400	3	1	192*256	192*256	1888.1	5000	99	4	192*256	384*512	49	40	2	201*230	177*256
1,5T Avanto / 1	8014.5	97	4	188x225	268x320	900	1400	3	1	192*256	192*256	2713.4	8910	93	4	225x225	230x256	49	40	2	180x230	158x256
1,5T Avanto / 1	8202.5	84	5	192x256	192x256	900	1400	3	1	192*256	192*256	1888.1	5000	99	4	192*256	384*512	46	40	3	158*230	125*192
3T Trio /1	6000	119	3	200x200	288x384	900	1380	2.2	1.1	211x211	192x192	2500	9000	125	3	200x200	192x256	28	20	1.2	137x230	175x320
3T Trio /1	6000	74	4	193x220	280x320	900	1380	2.2	1.1	211x211	192x192	1800	5000	93	4	193x220	224x256	27	20	1.5	173x230	182x256
3T Trio /1	6000	93	4	193*220	280*320	900	1380	2.2	1.1	211x211	192x192	1800	5000	93	4	193*220	224*256	27	20	1.5	173*230	182*256
3T Trio /1	6800	74	4	193*220	280*320	900	1380	2.2	1.1	211x211	192x192	1800	5000	93	4	193*220	224*256	27	20	1.5	173*230	182*256
3T Trio /1	6971	74	4	193*220	280*320	900	1380	2.2	1.1	211x211	192x192	1800	5000	93	4	193*220	224*256	27	20	1.5	151*230	160*256
